# Developing an ethical guideline for clinical teaching in Tehran University of Medical Sciences

**Published:** 2015-04-15

**Authors:** Akram Hashemi, Habibeh Yeketaz, Fariba Asghari

**Affiliations:** 1 PhD candidate, Department of Medical Education, Iran University of Medical Sciences, Tehran, Iran;; 2 MD, School of Medicine, Tehran University of Medical Sciences, Tehran, Iran;; 3 Associate professor, Medical Ethics and History of Medicine Research Center, Tehran University of Medical Sciences, Tehran, Iran.

**Keywords:** clinical teaching, ethical guideline, medical education

## Abstract

Clinical education is an essential part of medical trainees’ education process, and curriculum planners agree that it should be based on ethical standards and principles in the medical field. Nevertheless, no explained and codified criteria have been developed for ethics in clinical teaching. This study was aimed to develop an ethical guideline for medical students and teachers as the first and most important step in respecting patients' rights in educational centers.

The initial draft included the codes of ethics in clinical education and was developed based on library studies. Subsequently, it was improved through a qualitative study using semi-structured interviews and focus group sessions with medical students, patients, and medical teachers in educational hospitals affiliated to Tehran University of Medical Sciences. The improved draft was reviewed and validated by a medical expert panel to prepare the final draft.

The codes derived from this study included patients’ choices and rights in purely educational procedures, and special considerations for a) obtaining informed consent for educational procedures; b) performing procedures on deceased persons, patients under anesthesia and those lacking decision making capacity; c) educational visual recordings of the patients; and d) safety monitoring in clinical education.

The guideline developed in this study incorporates codes of ethics into clinical training. Therefore, in addition to providing efficient education, the interests of patients and their rights are respected, and the ethical sensitivity of learners in primacy of patients’ best interests will be preserved and enhanced.

## Introduction

Medical students encounter numerous educational opportunities and situations in the course of their training where ethical decision-making is important, and they have to respect the patients’ rights while considering their training needs (1). Studies have shown that 60 to 90 percent of patients agree to participate in the training of students and make their information available to them (2 - 4). Patients, however, believe that they are not provided with enough information on the trainees’ role in their medical care (5), and in many cases faculty members and students do not clarify their position to patients (6). All related ethical guidelines insist on obtaining informed consent for students performing clinical examinations on unconscious patients, and the majority of them consider a written consent as the preferred form. Research related to clinical examinations and interventions by students on anesthetized patients have shown that in about 25% of the cases no valid informed consent was obtained from the patients to perform such actions (7-10).

Numerous studies indicate that it is common to perform clinical interventions on deceased or dying individuals in educational-treatment centers of teaching hospitals (11) often without requesting permission from their families (12).

In the area of ​​ethics in medical education, issues related to the mutual rights of educators and learners have been discussed in ancient Iranian literature, while very little attention has been paid to patients' rights as educational subjects (13). Similarly, most topics in contemporary medical ethics pertain to health care and medical research, and ethics of medical education has rarely been discussed. Since most instances of modeling respect for patients’ rights occur at bedside, neglecting patients’ rights in the training process can seriously damage learners’ ethical sensitivity. 

It is essential to review the balance between providing outstanding health services and creating an appropriate educational environment for the students. In absence of appropriate clinical teaching, future physicians will not have the sufficient skills to provide the required medical services and may cause harm to the patients (7, 14-16). On the other hand, it is necessary to develop certain criteria for clinical education and the training process so as to respect the rights and interests of patients and maintain and enhance students’ ethical sensitivity. As a result, students will be guided to respect the rights of patients and prioritize their health and well-being while receiving efficient training (8, 16, 17). The present study aimed to develop an ethical guideline to safeguard patients’ rights in clinical teaching and guarantee ethical and humane bedside training.

## Method

This study was performed in three phases consisting of library study, qualitative research, and preparing the draft of the guideline. Initially, a comprehensive literature review was conducted by searching PubMed, Google, Google Scholar and university web pages using the following keywords: ethics, ethical guideline, clinical teaching, education, and patients’ rights. The above-mentioned web sources were also searched for papers and guidelines about patients’ rights in educational health centers and hospitals. Through this review and considering the cultural, social and religious conditions of the community, the initial draft of the Ethical Guideline on Patient Bedside Training in Clinical Educational Centers was developed in 17 clauses. The initial draft was reviewed by the academic advisors (four research members of the Medical Ethics and History of Medicine Research Center) and after considering their comments was approved as the preliminary guideline. Then, in the qualitative phase of the study, the draft was discussed in groups of stakeholders, including patients, clinical faculty members, students in different clinical grades, and medical ethics experts. Purposive sampling of stakeholders was performed in Tehran University of Medical Sciences until data saturation. The information of the participants was collected using interviews and focus group discussions. In total, the comments of 21 patients (equal numbers of patients in the emergency rooms and clinics and hospitalized patients) were collected through interviews. The comments of 21 students (6 trainees and 7 interns) were received through focus group discussions, and the comments of 6 specialized residents and 2 subspecialty residents were recorded through interviews. Similarly, the comments of 7 clinical professors who were in charge of educational affairs or had executive training positions, and 4 medical ethics experts in the Medical Ethics and History of Medicine Research Center were collected through interviews. Informed consent was obtained from participants for audio-recording of all individual and group interviews. In all the notes, a numerical code specific to each person was used rather than the names of participants. The collected comments were summarized and categorized separately for each article of the guideline. In the third stage, a consulting workshop attended simultaneously by 2 to 3 people from each stakeholder group was held. In this session, the initial draft of the Ethical Guideline on Patient Bedside Training and the comments gathered in the second stage were reviewed. The final changes were applied based on the collected feedback and the issues discussed in the consulting workshop, and the final version was prepared. Ultimately, the guideline was reviewed and edited.

## Results

After a comprehensive review of the literature related to patients’ rights in educational health centers and hospitals and considering the cultural, social, and religious conditions of the community, the initial draft of the Ethical Guideline on Patient Bedside Training in Clinical Educational Centers was developed. In the development of all sections of this guideline, compliance with common medical professional commitments was taken into account. Instances of these commitments included: improvement of professional competency and quality of care, honesty with patients, confidentiality, professional responsibilities, appropriate relations with patients, and justice.

This guideline contained special considerations on individuals lacking decision-making capacity, that is, patients with reduced consciousness, those suffering from intellectual disabilities or mental disorders, and children. 

In order to include such patients in educational-treatment programs, at least their verbal assent must be first obtained and then their parents’ or guardians’ consent should be sought (18-22). As for children, they should be provided with relevant information and consequently their parents’ or legal guardians’ consent should be obtained (18-22).

Some issues were also raised regarding learning procedures and examinations on patients under anesthesia. The general consensus was that in such cases, patients should be informed before anesthesia and their consent must also be obtained in writing (7-10).

Performing educational clinical interventions on deceased individuals was one of the cases discussed in developing this guideline. Such measures are aimed at training the students and improving their skills before they practice the procedures on live patients, and although they do not harm the deceased, it is essential to respect patients’ dignity in order to win and maintain their trust. It should be added that almost all reviewed literature considered it necessary to obtain written consent to perform such interventions on deceased patients.

The Society for Academic Emergency Medicine states that consent should be obtained from family members before practicing any procedures on freshly deceased bodies. The American Medical Association (AMA) Ethical Guideline advises that the educational procedures should not be performed on deseased patients in situations where it is not possible to obtain consent. The American Heart Association’s Emergency Cardiac Care committee suggests that informed consent should be obtained before using the newly dead for research or training purposes unless institutional guidelines specifically address circumstances under which consent is unnecessary (23-25).

In terms of obtaining consent to perform procedures on deceased patients, there was consensus among the participants in the present study; however, the disputed issue was the time to obtain the consent. Requesting such consent appears to be inhumane and perhaps unpractical in the distressing conditions experienced by the relatives of the deceased. It is preferable to seek patients’ consent under more normal circumstances as is the case for organ transplantation consent. This area of medical education ethics is rather challenging and requires further investigation and more extensive studies. Nevertheless, one can conclude that performing clinical interventions on deceased people is acceptable after obtaining consent from the deceased patients’ relatives provided that patients’ dignity is respected.

Regarding patients participating in training programs, the reviewed sources emphasized that the medical team should respect the patients’ rights to choose, and subjects have to be assured that they can withdraw from the training programs any time without compromising the health care they receive (2, 30, 31). As for making and using visual and audio recordings of patients, the reviewed literature considered patients’ consent necessary, although most sources agreed that verbal consent is sufficient in such cases (26, 27). Most members of the consultative workshop believed that written consent should be obtained for identifiable records due to the legal liabilities associated with such practices. In addition, the guideline developed in the present study stated that the photos or videos should be viewed by patients, and their potential request for discarding or not publishing them should be respected.

Some of the issues discussed in this guideline pertained to supervising students. The complex issues in this area can be summarized as follows:

1. The students should be trained on moulages, standardized patients or their classmates prior to entering clinical courses. Although the true learning of skills is achieved when practiced on real patients, the latter could be harmed or injured in the process.
2. To eliminate or reduce the risks for patients, the learners' participation in clinical interventions can be conditional on direct and appropriate supervision of a trained senior. The senior in charge of the medical team would naturally be responsible for monitoring the students and possible damage and harm to the patients.
3. Issues to be considered in training for clinical skills using patients include reviewing the risks associated with students practicing the interventions on patients, informing patients about participation in clinical interventions and obtaining their consent, and fair distribution of the medical training load among different patients.

Through the stages described above, the final draft of the guideline was finalized. This draft included an introduction, ethical principles based on which the guideline was developed, codes (including 15 items presented in Table 1) and the glossary. 

**Table 1:** A brief review of an ethical guideline for clinical teaching

1	Patients should be well informed about the role of medical trainees in their care process.
2	All members of the medical team have to introduce themselves and explain their roles and responsibilities to patients.
3	All members of the medical team should wear ID cards.
4	All members of the medical team should respect patients’ autonomy in receiving "purely educational" procedures.
5	Prior to performing educational procedures on incompetent patients, all members of the medical team should ask for their guardians’ permission and respect the patients’ refusal to cooperate.
6	Prior to performing anesthesia, the medical team have to obtain patients’ written consent for examinations with "purely educational" purposes.
7	For clinical interventions with "purely educational" purposes on deceased patients, the medical team have to respect the dead patients’ dignity and obtain their (families’) consent.
8	The senior in charge of the medical team has to appropriately make sure that students have acquired the basic clinical skills, and then allow them to perform the procedures on patients under the senior’s supervision.
9	Based on the intervention risks and the skills and experience level of the students, the senior in charge of the medical team has to appropriately supervise the quality of clinical interventions offered by the students.
10	The medical team should use the patients’ information in unnamed forms in morning report sessions and educational conferences, and should otherwise ask for the patients’ consent.
11	The medical team have to respect patient privacy in "purely educational” programs in terms of patients’ participation in the class, morning reports or students’ exams, and make sure not to pressure them in any way.
12	The medical team have to ask for the patients’ consent for any visual recordings of them, and if such recordings are identifiable, written consent will have to be obtained.
13	The medical team have to inform the patients of their right for presence of a chaperone in any sensitive examinations, or examinations by persons of the opposite gender.
14	The medical team should respect the child patients’ rights for presence of their parents.
15	The senior in charge of the medical team has to make sure that appropriate communication is conducted between patients and the trainees involved in their care.

## Discussion

The purpose of developing this guideline was to create a balance between training the next generation of physicians and respecting the rights of patients who participate altruistically in the process of medical education.

In a number of medical universities in other countries, such as Queen's University of Canada, and the University of Sydney, guidelines for ethics in clinical teaching have been developed, and professors and students in different grades are required to follow them (28, 29).

The ethical guideline developed in this study has very much in common with most other guidelines, although it is different in some respects. The reason is apparently the divergence in cultures and the different regulations governing the health systems as well as universities. Among these issues, monitoring patients’ safety in clinical teaching can be mentioned.

In some universities, due to strict observation of patient safety criteria and serious legal responsibilities of the physician in charge in case of damage to patients’ health, there is close monitoring of the students’ performance. As a result, there has been no need to explain the subject extensively in their ethical guidelines for training at patients’ bedside. For example in the ethical guideline of Capital & Coast District Health Board (CCDHB) in New Zealand and Queen’s University of Canada, seniors’ responsibility for supervision on patient safety has been mentioned briefly (28, 30). In Iran, however, there are shortcomings in patient safety monitoring in hospitals, and we have therefore discussed senior supervision and assessment of the students’ skills in our ethical guideline in detail. 

Another prominent point in our guideline is making visual recordings of patients for use in training. This issue has not been discussed in guidelines of universities of the US, UK and Malaysia where specific national guidelines are in place for audio visual recordings for service, research and education (26, 27, 31).

In the case of our ethical guideline, the topic of images and videos from patients was not related to clinical teaching. Nevertheless, it was necessary to examine the subject in detail due to the importance of using photos and videos of the patients in medical education and lack of ethical and legal standards within the health care system in this regard. Another issue that was extensively discussed in our guideline was the necessity of informing patients about the specific rules of educational hospitals and the role of trainees in providing services in these settings. There are serious deficiencies in providing the patients with information on the general regulations of health care centers and institutions, the specific rules of teaching hospitals, the hierarchy in providing services, and the role and duties of medical students at different levels. Thus, the above issues were particularly emphasized in our ethical guideline.

Our ethical guideline for bedside teaching is helpful for medical trainees of all levels and can play an important role in institutionalization of medical ethics in clinical education. Additionally, observance of the issues discussed in this guideline can be considered a sort of practical and applied training in medical ethics during clinical courses. On the other hand, it leads to winning the patients’ trust through respecting and observing their rights. As a result, patients will be more cooperative during bedside training, and the students’ educational level and respect for patients’ rights will be improved.

Developing this guideline and its implementation can unify the learners’ professional behavior in dealing with patients and reduce the likelihood of various forms of abuse resulting from patients' lack of knowledge. After compiling and communicating the ethical instructions on clinical teaching to the affiliated educational hospitals, they are expected to design and execute a comprehensive plan for informing the patients about their rights and brief the students and professors on the guideline. Although the ethical guidelines on clinical teaching are not considered a legal set of instructions, they can be used to prevent possible abusive behaviors or violation of patients’ rights in educational hospitals.

In addition to medicine, a number of other fields of medical sciences such as dentistry, clinical pharmacy, nursing and rehabilitation medicine offer some clinical teaching during their educational courses. The current guideline can serve as a model for developing an ethical framework for clinical practice in such fields. On one hand, the guideline is beneficial for the students in these fields and on the other hand, it will be a safeguard to protect the patients’ rights in educational centers.

Regarding clinical interventions on deceased persons, it seems necessary to do more extensive studies to investigate the views of community members and the possible drawbacks of obtaining informed consent.

Although developing this guideline required considerable effort, it is not beyond criticism and before its implementation in universities, the comments of all the professors and students must be taken into consideration. 
